# Analysis of the relationship between sleep-related disorder and systemic immune-inflammation index in the US population

**DOI:** 10.1186/s12888-023-05286-7

**Published:** 2023-10-24

**Authors:** Kaisaierjiang Kadier, Diliyaer Dilixiati, Aikeliyaer Ainiwaer, Xiaozhu Liu, Jiande Lu, Pengfei Liu, Mierxiati Ainiwan, Gulinazi Yesitayi, Xiang Ma, Yitong Ma

**Affiliations:** 1 Department of Cardiology, First Affiliated Hospital of Xinjiang Medical University, State Key Laboratory of Pathogenesis, Prevention and Treatment of High Incidence Diseases in Central Asia, Urumqi, China; 2 Department of Urology, First Affiliated Hospital of Xinjiang Medical University, Urumqi, China; 3 Department of Cardiology, The Second Affiliated Hospital of Chongqing Medical University, Chongqing, China

**Keywords:** Systemic immune-inflammation index, Sleep-related disorder, Obstructive sleep apnoea, Daytime sleepiness, Sleep problems, Sleep duration, NHANES

## Abstract

**Background:**

The association between sleep-related disorders and inflammation has been demonstrated in previous studies. The systemic immune-inflammation index (SII) is a novel inflammatory index based on leukocytes, but its relationship with sleep-related disorder is unclear. We aimed to investigate the relationship between sleep-related disorder and SII in a nationally representative nonhospitalized sample.

**Methods:**

Data were obtained from the 2005–2008 National Health and Nutrition Examination Survey (NHANES). Exposure variables included self-reported sleep-related disorders, such as sleep duration, sleep problems, high risk of OSA, and daytime sleepiness. SII and other traditional markers of inflammation were considered as outcome variables, including platelet-to-lymphocyte ratio (PLR) and neutrophil-to-lymphocyte ratio (NLR). Multiple linear regression models were employed to examine the correlation between sleep-related disorders and inflammatory markers. Subgroup interactions were analyzed using likelihood ratio tests, and nonlinear relationships were explored by fitting restricted cubic splines.

**Results:**

A total of 8,505 participants were enrolled in this study. Overall, sleep-related disorders were found to have a stronger association with SII compared to the PLR and NLR. The results of multiple linear regression analysis revealed that participants who experienced sleep problems (β: 21.421; 95% CI 1.484, 41.358), had symptoms of OSA (β: 23.088; 95% CI 0.441, 45.735), and reported daytime sleepiness (β: 30.320; 95% CI 5.851, 54.789) exhibited a positive association with higher SII. For the analysis of other inflammatory markers, we only found that daytime sleepiness was associated with increased NLR levels (β: 0.081; 95% CI 0.002, 0.159).

**Conclusion:**

Sleep problems, symptoms of OSA, and daytime sleepiness were found to have a positive association with the SII in US adults. However, further prospective studies are necessary to establish whether there is a causal relationship between these factors.

**Supplementary Information:**

The online version contains supplementary material available at 10.1186/s12888-023-05286-7.

## Introduction

Sleep is an indispensable physiological function, and everyone spends one-third of their lifetime asleep. With the increasing pace of life and social pressure, an increasing number of people are afflicted with sleep disorders. For example, less than half of Americans sleep as long as recommended by the National Sleep Foundation [1], and sleep disorders affect approximately one-third of the adult population [2]. The Global Prevalence and Burden Assessment of Obstructive Sleep Apnea (OSA) states that an estimated 936 million adults worldwide have mild to severe OSA, with the United States ranking second among those affected [3]. A growing body of research indicates that sleep disorders can exert a significant impact on various facets of human health, potentially giving rise to a range of physical ailments stemming from psychological factors [4]. Moreover, these complex psychosomatic disorders are associated with significant economic and social burdens, indicating that they are likely to become a growing global concern in the coming years.

Changes in sleep and circadian rhythms can dynamically adjust the immune system through physiological processes such as immune cell redistribution and inflammatory factor production [5]. Sleep disorders are associated with most diseases and are thought to disrupt physiological processes that regulate the immune system, resulting in abnormally increased inflammatory responses that drive disease progression [6, 7]. In addition, the relationships among the above processes are considered bidirectional [8]. The results of a meta-analysis indicated that sleep disorders and long sleep duration are associated with an increase in systemic inflammatory markers such as C-reactive protein [9]. In addition, a cross-sectional NHANES-based study also consistently observed that blunted rest-activity circadian rhythms were associated with increased leukocyte inflammatory indices derived from routine blood tests [10]. At the same time, Endogenous inflammation and pathogen infection disrupt normal sleep patterns by transmitting cytokines across the blood–brain barrier to the central nervous system through neuromodulation and humoral mediators [5]. For example, the production of inflammatory cytokines by TLR4-stimulated monocytes at bedtime has been found to predict the sleep efficiency and duration of slow-wave sleep in patients with rheumatoid arthritis [11]. Therefore, it is imperative to clarify the effects of changes in sleep behavior and the risk of sleep disorders on systemic inflammatory status. This will help to establish the connection between sleep and other inflammatory diseases, and enable the monitoring of healthy sleep patterns to enhance overall health.

The systemic immune-inflammation index (SII) is an inflammatory marker first proposed by Hu et al. that can be obtained based on blood leukocyte parameters [12]. The SII combines the combined manifestations of three types of inflammatory cells, including platelets, neutrophils and lymphocytes, which can reflect the systemic immune response and inflammatory level while having inexpensive, stable and effective characteristics [13]. Increasing evidence suggests that SII, neutrophil-to-lymphocyte ratio (NLR) and platelet-to-lymphocyte ratio (PLR) are markers of psychiatric disease severity derived from peripheral blood cell counts [14, 15]. Investigations into the psychopathological status of survivors of COVID-19 have revealed that long-standing systemic inflammatory status can be reflected by inflammatory parameters such as SII and is closely related to anxiety and somatization and sleep disorders [16, 17]. However, another study only reported higher SII levels in long-COVID patients, but no significant association was found with psychopathological items such as sleep disorders [18]. In a recent retrospective cohort study, Topuz et al. first reported that the SII was strongly associated with OSA severity and performed better than the NLR and PLR [19]. As far as we know, the study of sleep and SII is still in its infancy, and relationship between the sleep duration and sleep disorders and SII has not been clarified.

Inspired by the background of previous studies, the aim of our study was to explore the relationship between sleep-related disorder and SII through a nationally representative complex sampling sample from the National Health and Nutrition Examination Survey (NHANES) with performance of additional analyses of the traditional inflammatory indicators NLR and PLR.

## Materials and methods

### Study population

The NHANES provides information about the health and nutritional status of noninstitutionalized civilians in the United States, based on a large nationwide cross-sectional survey. NHANES uses a complex, multistage probability sampling approach to obtain a representative sample of individual composition to investigate the overall national population. In this study, we integrated data from the 2005–2006 and 2007–2008 surveys because these two cycles contained the most comprehensive sleep disorder questionnaires available to date. In a standard procedure, participants completed home interviews and subsequently underwent physical examinations and biochemical sample collections at a mobile examination center (MEC). The NHANES survey project was approved by the National Center for Health Statistics Institutional Review Board and Ethics Review Board, and all participants provided written informed consent. This study was drafted according to the Strengthening Reporting of Observational Studies in Epidemiology (STROBE) Guidelines for Reporting Cross-sectional Studies [20].

**Fig. 1:**
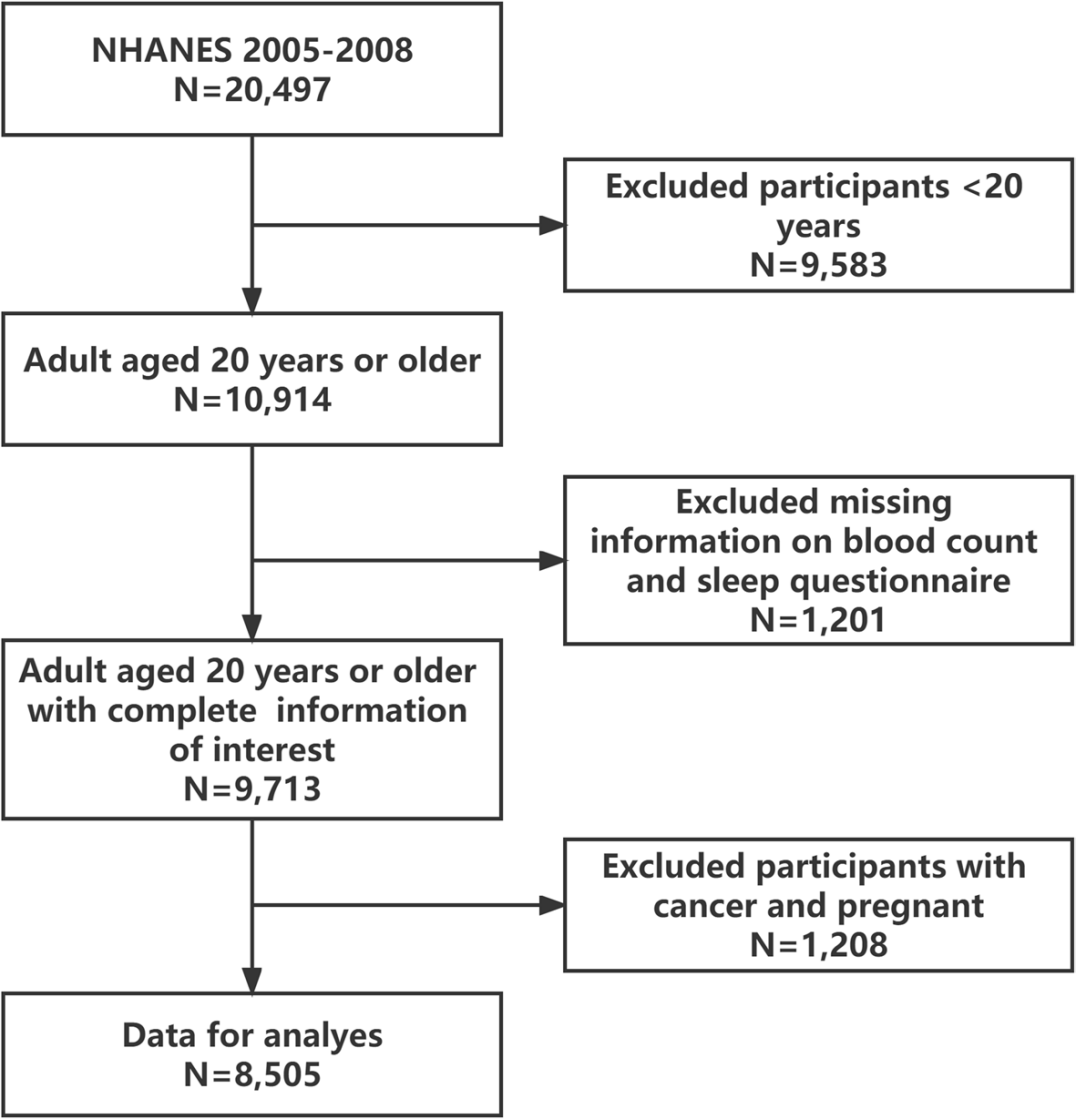
Flowchart of the participants selection from NHANES 2005–2008. NHANES, National Health and Nutrition Examination Survey

Exclusion criteria for participants we analyzed were (i) age < 20 years, (ii) missing or incomplete data on complete blood count and sleep questionnaires, (iii) having cancer, and (iv) being pregnant. In 2005–2008, 2 survey cycles were completed, and a total of 20,497 individuals were first enrolled; 8,505 participants were included in our final analysis after exclusion of subjects aged < 20 years (9,583), missing complete blood count (1,085) and sleep questionnaire data (116), having cancer (864), and being pregnant (344) (Fig. 1).

### Definition of the sleep-related disorder

Questions on sleep are new to NHANES in 2005–2008. Adapted from the Sleep Habits Questionnaire for the Sleep Heart Health Study, this section contains questions about sleep habits and disorders [21]. We conducted a study on sleep-related disorders and examined several exposure variables, including sleep duration, sleep problems, high risk of OSA, and daytime sleepiness.

Sleep duration was analyzed separately as categorical outcome variables. Sleep duration < 7 h/night: insufficient sleep, 7–9 h/night: normal sleep, or > 9 h/night: excessive sleep [22]. Having sleep problems [23] was defined as having self-reported frequency greater than or equal to 5 times per month for the following questions: “How often do you wake up during night/have trouble falling asleep/wake up too early in morning?”; or affirmative answers to the following questions: “Have you ever been told by a doctor or other health professional that you have a sleep disorder or trouble sleeping?”. Daytime sleepiness [23] was also defined according to self-report and was considered to be present if the response frequency was more than or equal to 5 times per month when answering the following questions: “How often did you feel excessively sleepy/feel unrested during the day, no matter how much sleep you get?”.

High-risk for OSA was defined according to the STOP screening questionnaire for OSA [24]. This questionnaire defined high-risk for OSA as having two or more of the four symptoms recommended by the American Academy of Sleep Medicine for OSA screening, including hypertension, snoring, cessation of breathing, and daytime sleepiness [25]. To achieve this classification, snoring and cessation of breathing are obtained by answering “5 or more nights/week” to any of the following questions from the NHANES Sleep Disorders Questionnaire: “How often did you stop breathing, gasp, or snore while you slept in the past year?”. In addition to the previously described method, we directly classified participants who were definitively diagnosed with sleep apnea by a physician as being at high-risk for OSA.

### Definition of the systemic immune-inflammation index

Lymphocyte, neutrophil and platelet counts were obtained by performing a complete blood count on blood specimens with a Beckman Coulter automated blood analyzer in an MEC and were expressed as × 10^3^ cells/µL. According to previous studies [12, 26], SII was defined as follows: SII = Platelet count*Neutrophil count/Lymphocyte count. To more fully assess the association between sleep-related disorders and SII, we further assessed PLR and NLR, which are commonly used blood inflammatory markers in clinical studies. In this study, SII, PLR and NLR were designed as outcome variables.

### Covariates

According to previous studies [23, 27], we included covariates in the analysis given the confounding of study results by other factors. We included the following covariates based on NHANES interview and examination, laboratory and questionnaire data: age, sex, race, education level, poverty-income ratio (PIR), body mass index (BMI), smoking status, alcohol consumption status, recreational activities, hypertension, diabetes, cardiovascular disease (CVD) and taking immunosuppressants. Socioeconomic factors were assessed and collected during the home interview. Based on the original survey records, PIR was categorized into three groups: < 1.3, 1.3 – 3.5, and > 3.5. It was observed that lower PIR was linked to higher levels of poverty. Behavioral factors were obtained through self-reports. A never smoker is an individual who has never smoked more than 100 cigarettes in their lifetime. Former smokers are defined as those who have smoked more than 100 cigarettes in their lifetime but have since quit smoking. Current smokers are individuals who have smoked at least 100 cigarettes in their lifetime and continue to smoke on some days or every day. Alcohol consumption was categorized as: Never drinkers: had < 12 drinks in lifetime. Former drinkers: had ≥ 12 drinks in 1 year and did not drink last year, or did not drink last year but drank ≥ 12 drinks in lifetime. Current mild-moderate drinkers: ≤ 1 drink per day for women or ≤ 2 drinks per day for men on average over the past year. Current heavier drinkers: > 1 drink per day for women or > 2 drinks per day for men on average over the past year. Recreational activity was defined based on individuals' self-reported levels of activity over a period of 30 days. BMI was measured at an MEC using standardized protocols and categorized into two groups: ≥ 30 (obese) and < 30 (non-obese). Participants who self-reported a medical diagnosis of heart failure, angina, coronary heart disease, heart attack, or stroke, as confirmed by a physician, were categorized as having CVD. The definition of hypertension is a diagnosis made by a healthcare professional, based on an average blood pressure reading of ≥ 130/80 mmHg or the use of hypertension medications. Diabetes was defined as a diagnosis made by a physician or other healthcare professional, glycated hemoglobin (%) greater than 6.5, random blood glucose (mmol/L) equal to or greater than 11.1, or use of diabetes medication or insulin. Obtaining information about the participant's use of immunosuppressants was done through a home interview conducted by the staff. They identified the participant's prescription medication sheet and kit information. Detailed definitions of covariates and remaining information are provided in Supplementary Table 1.

### Statistical analysis

Descriptive statistics were calculated to describe the characteristics of the participants according to the classification of SII quartiles. Continuous variables are presented as the weighted mean ± standard deviation (SD), while categorical variables are presented as weighted percentages (95% confidence interval, 95% CI) and were compared using one-way ANOVA and the Rao-Scott chi-square test, respectively. Multiple linear regression was employed to assess the relationship between various inflammatory parameters as dependent variables and sleep-related disorders as independent variables. Beta coefficients and 95% CIs were calculated. Model 1 was crude (not adjusted). Model 2 was adjusted for age, sex, and race. Model 3 was adjusted for age, sex, race, education level, PIR, BMI, smoking status, alcohol consumption status, recreational activities, hypertension, diabetes, CVD and taking immunosuppressants. At the same time, subgroup analyses within fully adjusted models were performed stratified by age (< 60/ ≥ 60 years), sex (male/female), and race (non-Hispanic white/non-Hispanic black/other), and multiplicative interactions were assessed using likelihood ratio tests. Restricted cubic splines with 3 knots at the 10th, 50th, and 90th percentiles were used to explore the nonlinear relationships of sleep exposure variables and inflammatory indicator outcomes in the fully adjusted model.

Because it is a complex sampling design for the national population, we considered 2-year MEC weights (WTMEC2YR), sampling units (SDMVPSU) and strata (SDMVSTRA) in all analyses [28]. In this case, new 4-year weights could be calculated by dividing the 2-year weights by two.

We used the MissForest package [29] to impute missing values for covariates, whether continuous or categorical. The number and percentage of missing covariate data are shown in Supplementary Table 2. Sensitivity analyses were performed as follows: (1) included only participants with complete data and excluded those with missing covariates; (2) excluded individuals who had recently experienced symptoms of infection, such as head or chest colds, gastric or intestinal diseases accompanied by vomiting or diarrhea, influenza, pneumonia, or ear infections; and (3) Furthermore, we adjusted for OSA symptoms when examining the relationship between daytime sleepiness and inflammatory markers. Statistical analysis was conducted using R software (version 4.1.3; https://www.R-project.org) with a complex sampling module. All statistical analysis P values were from two-sided tests, and the results were deemed statistically significant at P values less than 0.05.

## Results

### Participant characteristics

A total of 8,505 samples were included in this study, weighted to represent 180 million U.S. noninstitutionalized adult populations. The baseline participant characteristics are listed in Table 1.The mean age of the population surveyed was 45.74 ± 0.39 years, while 50.50% (95% CI: 46.08%, 54.91%) were female and 70.13% (95% CI: 60.31%, 79.96%) were non-Hispanic White. For the distribution of sleep duration of participants, 37.48% (95% CI: 34.51%, 40.45%) slept less than 7 h, and 2.17% (95% CI: 1.70%, 2.63%) slept more than 9 h. Moreover, For sleep-related disorder as defined in this study, sleep problems, high-risk for OSA and daytime sleepiness accounted for 41.40% (95% CI: 37.20%, 45.60%), 43.16% (95% CI: 38.97%, 47.34%) and 29.13 (95% CI: 26.27%, 32.00%) of the population, respectively.

**Table 1:** General characteristics of included participants (*n* = 8,505) according to systemic immune-inflammation index quartiles in the NHANES 2005–2008

Characters	Overall (n = 8,505)	Q1 (*n* = 2,127) (13.750, 364.219)	Q2 (*n* = 2,124) (364.219, 506.647)	Q3 (*n* = 2,127) (506.647, 708.923)	Q4 (*n* = 2,127) (708.923, 5137.500)	*P*-value
**Age, year**	45.74 ± 0.39	44.69 ± 0.57	45.72 ± 0.41	45.88 ± 0.44	46.48 ± 0.66	0.031
**Sex**						< 0.0001
Male	49.50(45.43–53.58)	58.03(55.96–60.10)	49.63(46.76–52.50)	50.40(48.30–52.50)	41.39(39.35–43.42)	
Female	50.50(46.08–54.91)	41.97(39.90–44.04)	50.37(47.50–53.24)	49.60(47.50–51.70)	58.61(56.58–60.65)	
**Race**						< 0.0001
Mexican American	8.62( 6.96–10.29)	8.76(6.88–10.64)	8.40(6.52–10.28)	8.94(6.78–11.11)	8.40(6.17–10.63)	
Non-Hispanic Black	11.15( 8.98–13.32)	19.82(15.28–24.36)	10.05( 7.35–12.74)	8.63( 6.40–10.85)	7.51( 5.49–9.52)	
Non-Hispanic White	70.13(60.31–79.96)	60.42(54.42–66.41)	71.16(66.59–75.72)	72.44(68.17–76.71)	74.96(70.39–79.52)	
Other Hispanic	4.33( 2.90–5.76)	4.27(2.83–5.70)	4.60(2.76–6.43)	4.49(2.84–6.14)	3.96(2.50–5.41)	
Other race	5.76( 4.69–6.84)	6.74(4.78–8.69)	5.80(4.15–7.44)	5.50(3.84–7.16)	5.18(3.64–6.72)	
**Education**						0.352
Less than hight school	18.86(16.39–21.32)	19.85(17.13–22.56)	18.74(16.22–21.26)	18.66(15.79–21.54)	18.34(15.44–21.24)	
Hight school	24.98(22.20–27.76)	22.77(19.49–26.04)	24.30(22.15–26.45)	25.39(22.90–27.87)	27.07(25.12–29.02)	
Above hight school	56.17(50.81–61.52)	57.39(53.74–61.03)	56.96(53.71–60.21)	55.95(51.74–60.16)	54.59(50.75–58.44)	
**Poverty-income ratio**						0.016
< 1.3	17.97(16.06–19.89)	19.12(16.58–21.65)	15.96(14.28–17.65)	17.15(14.73–19.57)	19.78(17.24–22.31)	
1.3–3.5	38.34(35.03–41.65)	40.24(36.59–43.89)	37.37(34.35–40.38)	37.01(33.65–40.37)	41.21(37.21–45.21)	
> 3.5	43.69(38.03–49.35)	40.64(36.11–45.17)	46.67(43.14–50.20)	45.84(41.06–50.62)	41.21(37.21–45.21)	
**BMI (kg/m**^**2**^**)**						< 0.0001
< 30	65.95(60.79–71.11)	72.39(69.81–74.97)	66.89(64.16–69.61)	63.74(61.22–66.27)	61.87(58.57–65.18)	
≥ 30	34.05(30.22–37.87)	27.61(25.03–30.19)	33.11(30.39–35.84)	36.26(33.73–38.78)	38.13(34.82–41.43)	
**Smoking status**						0.045
Now	24.03(21.22–26.83)	21.64(19.03–24.26)	21.70(18.85–24.56)	25.24(22.74–27.75)	27.06(23.90–30.21)	
Former	23.57(21.23–25.91)	24.50(22.13–26.86)	23.78(21.65–25.91)	23.15(20.86–25.44)	23.01(20.77–25.26)	
Never	52.40(47.82–56.98)	53.86(51.31–56.41)	54.52(51.75–57.29)	51.61(48.34–54.88)	49.93(45.69–54.17)	
**Alcohol consumption status**						0.472
Never	11.90(10.39–13.40)	12.85(11.05–14.65)	11.54( 9.12–13.96)	11.87( 9.76–13.98)	11.48( 9.74–13.21)	
Former	16.55(14.08–19.02)	16.25(14.30–18.21)	16.95(14.34–19.56)	16.72(14.26–19.18)	16.24(14.16–18.33)	
Mild-Moderate	33.18(29.79–36.56)	33.50(30.71–36.30)	34.28(31.48–37.07)	33.78(30.94–36.61)	31.25(28.46–34.05)	
Heavy	38.38(34.64–42.11)	37.39(34.19–40.60)	37.24(34.02–40.46)	37.63(34.64–40.63)	41.03(38.32–43.73)	
**Recreational activitie**						< 0.0001
Vigorous	31.45(28.19–34.70)	36.95(33.10–40.80)	33.15(30.07–36.23)	30.27(27.78–32.76)	26.37(23.10–29.65)	
Moderate	28.03(25.11–30.95)	26.16(23.62–28.69)	28.93(26.47–31.39)	28.72(25.65–31.79)	28.05(25.51–30.59)	
Inactive	40.52(35.49–45.55)	36.89(32.90–40.88)	37.92(34.09–41.74)	41.01(37.74–44.28)	45.58(40.99–50.16)	
**CVD**	7.70( 6.55–8.85)	8.19(7.06–9.32)	6.25(5.15–7.35)	7.26(5.75–8.76)	9.14(7.60–10.69)	0.011
**Hypertension**	46.70(42.31–51.10)	44.11(40.71–47.52)	44.78(41.99–47.56)	48.31(45.69–50.93)	49.12(46.74–51.51)	0.028
**Diabetes**	9.95( 8.79–11.11)	10.95(9.23–12.66)	8.55(7.26–9.84)	9.89(8.63–11.16)	10.53(8.74–12.33)	0.098
**Taking immunosuppressants**	0.71( 0.47–0.95)	0.86(0.29–1.43)	0.44(0.08–0.80)	0.39(0.07–0.72)	1.17(0.55–1.78)	0.079
**Sleep duration**						0.301
< 7 h/night	37.48(34.51–40.45)	39.85(36.30–43.40)	36.92(33.85–39.98)	36.07(33.47–38.67)	37.45(34.76–40.14)	
7–9 h/night	60.35(54.37–66.33)	58.35(54.95–61.75)	61.15(58.02–64.29)	61.54(58.75–64.32)	60.08(57.48–62.67)	
> 9 h/night	2.17( 1.70–2.63)	1.80(1.10–2.51)	1.93(1.34–2.52)	2.39(1.62–3.16)	2.48(1.88–3.07)	
** Sleep problems**	41.40(37.20–45.60)	37.77(35.06–40.47)	40.47(37.92–43.01)	40.23(38.12–42.33)	46.51(43.94–49.09)	< 0.001
**OSA symptoms**	43.16(38.97–47.34)	38.83(36.70–40.97)	40.62(37.77–43.47)	45.32(43.01–47.63)	47.05(44.28–49.82)	< 0.0001
**Daytime sleepiness**	29.13(26.27–32.00)	26.48(24.29–28.68)	27.44(25.21–29.67)	28.09(26.02–30.16)	34.01(31.42–36.60)	< 0.0001

*Abbreviations: BMI* Body mass index, *CVD* Cardiovascular Disease, *OSA* Obstructive sleep apnea

Continuous variables are presented as the weighted mean ± standard deviation, and were compared using the weighted one-way ANOVA test

Categorical variables are presented as weighted percentages (95% confidence interval), and were compared using the weighted Rao-Scott chi-square test

**Fig. 2:**
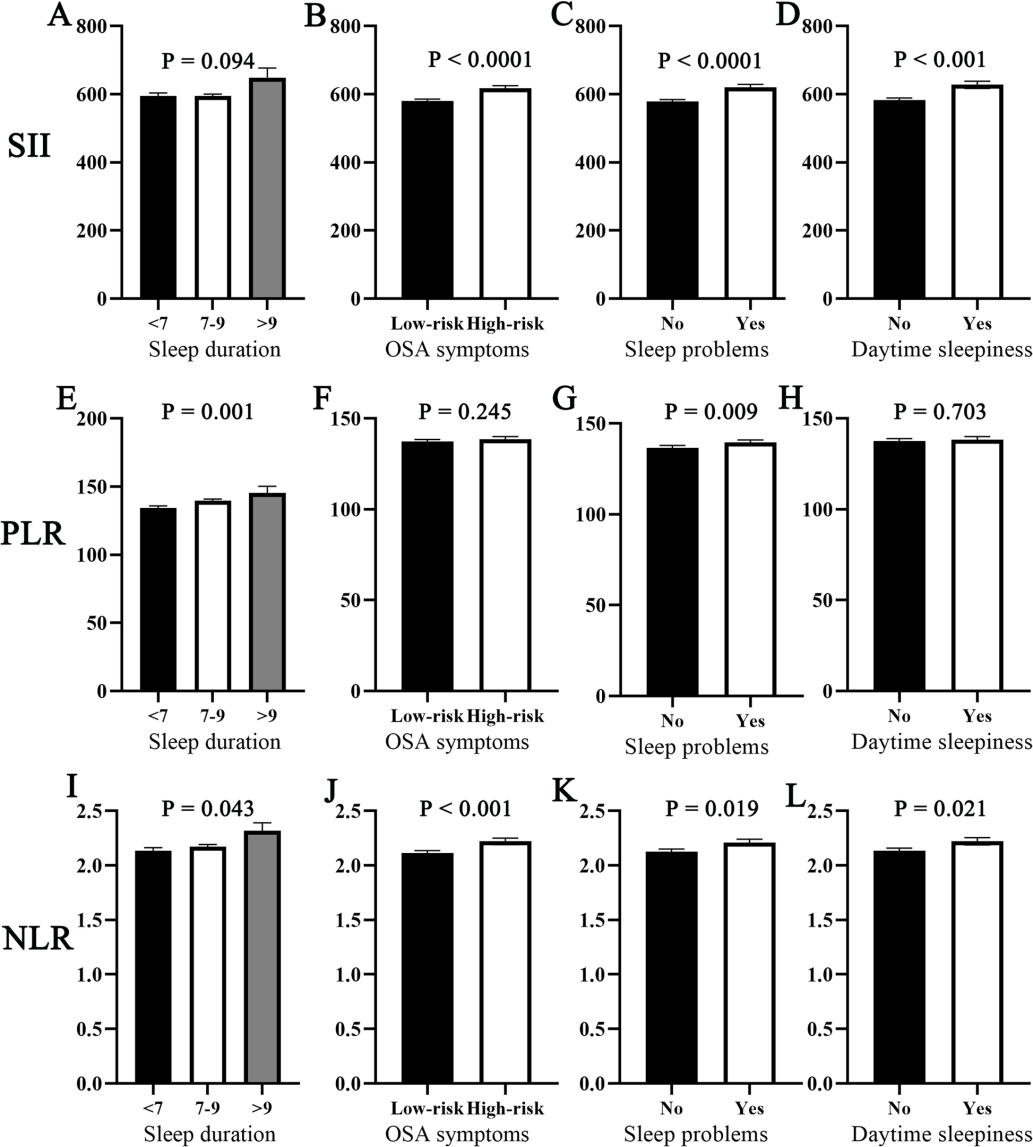
Differences in SII, PLR and NLR inflammatory markers among different sleep-related disorder exposures. **A** SII and sleep duration; (**B**) SII and OSA symptoms; (**C**) SII and sleep problems; (**D**) SII and daytime sleepiness; (**E**) PLR and sleep duration; (**F**) PLR and OSA symptoms; (**G**) PLR and sleep problems; (**H**) PLR and daytime sleepiness; (**I**) NLR and sleep duration; (**J**) NLR and OSA symptoms; (**K**) NLR and sleep problems; (**L**)NLR and daytime sleepiness. **P** value was calculated by weighted T-test or one-way ANOVA test. SII, systemic immune-inflammation index; PLR, platelet-to-lymphocyte ratio; NLR, neutrophil-to-lymphocyte ratio. OSA, obstructive sleep apnea

According to the SII quartiles, we observed differences in age, sex, race, PIR, BMI, smoking status, recreational activities, hypertension, CVD, sleep problems, high-risk for OSA and daytime sleepiness. It is crucial to highlight that the percentage of individuals living in poverty has risen within both the highest and lowest SII categories. At the same time, Fig. 2 demonstrates differences in SII, PLR and NLR inflammatory markers among different sleep-related exposure status groups. We found that the SII differed among the sleep problem, high-risk for OSA, and daytime sleepiness groups. We obtained similar results in NLR but differed from the former in sleep duration. Interestingly, the results of PLR were unique and differed only between the sleep duration and sleep problem groups.

### Association between sleep duration and SII 

**Fig. 3:**
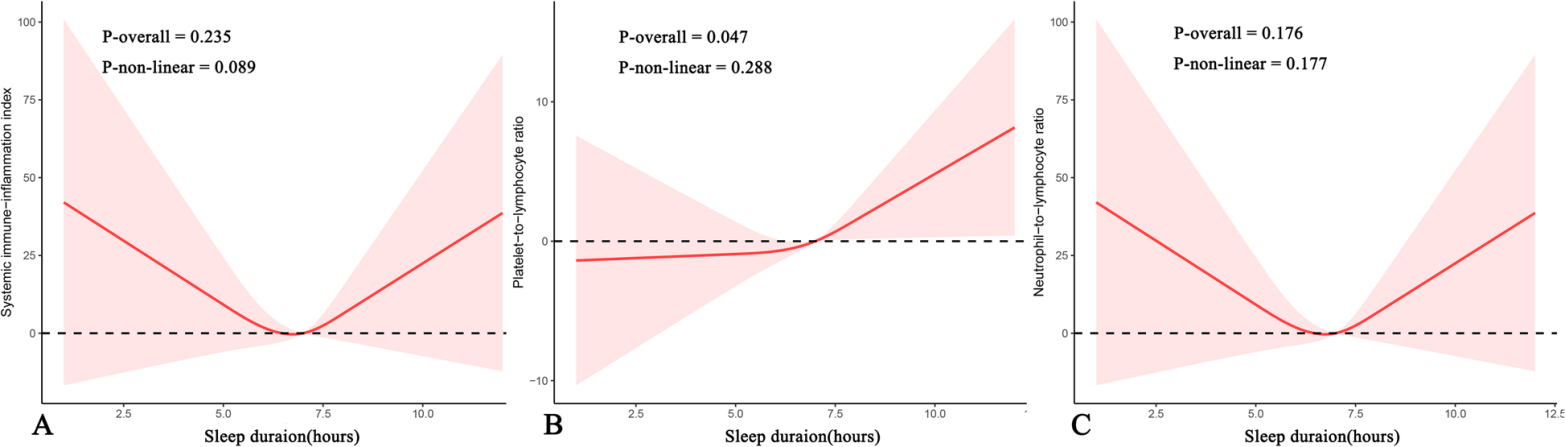
Nonlinear relationship between sleep duration and leukocyte inflammatory markers by restricted cubic spline fitting. **A** sleep duration and systemic immune-inflammation index; (**B**) sleep duration and platelet-to-lymphocyte ratio; (**C**) sleep duration and neutrophil-to-lymphocyte ratio. Adjusted for age, sex, race, education level, PIR, BMI, smoking status, alcohol consumption status, recreational activities, hypertension, diabetes, CVD and taking immunosuppressants

Table 2 presents the relationships between the sleep duration classification and SII and the other inflammatory markers. Compared to normal sleep duration, no association was found between both insufficient sleep and excessive sleep and SII. In further analyses of PLR and NLR, linear regression results revealed a negative association between insufficient sleep and PLR, and a positive association between excessive sleep and NLR. However, it is important to note that these associations disappeared in fully adjusted models. By applying a cubic spline curve, we attempted to explore a potential U-shaped association between sleep duration and SII and NLR. However, after conducting significance testing, it was determined that there is no significant relationship between the two variables. In addition, it was found that sleep duration had a linear association with PLR. The Fig. 3 illustrates the specific results.

**Table 2:** Weighted linear regression coefficients (β) and 95% confidence intervals for the association between sleep duration and SII and inflammatory markers: The United States, 2005 to 2008

Exposure	Outcome	Categories	Model 1^a^ β (95% CI), P- value	Model 2^b^ β (95% CI), P- value	Model 3^c^ β (95% CI), P- value
Sleep duration	SII	7–9 h/night	Reference	Reference	Reference
		< 7 h/night	0.038(-16.479, 16.555) *P *= 0.996	12.165(-4.594, 28.925) *P* = 0.147	1.229(-17.940, 20.399) *P* = 0.884
		> 9 h/night	54.748(-0.861,110.357) *P* = 0.053	52.542(-0.858,105.941) *P* = 0.053	35.656(-29.280,100.592) *P* = 0.235
	PLR	7–9 h/night	Reference	Reference	Reference
		< 7 h/night	**-5.311(-8.397,-2.224)** *P* = 0.001	**-4.848(-8.074,-1.622)** *P* = 0.005	-3.227(-6.918, 0.465) *P* = 0.078
		> 9 h/night	5.694(-3.956,15.344) *P *= 0.237	3.817(-6.155,13.788) *P* = 0.437	5.426(-6.092, 16.944) *P* = 0.302
	NLR	7–9 h/night	Reference	Reference	Reference
		< 7 h/night	-0.038(-0.085,0.010) *P* = 0.115	0.003(-0.045,0.050) *P* = 0.913	-0.024(-0.077, 0.029) *P* = 0.321
		> 9 h/night	**0.145(0.003,0.288)** *P* = 0.046	**0.161(0.028,0.295)** *P* = 0.020	0.089(-0.072, 0.251) *P* = 0.232

*Abbreviation: CI* Confidence interval, *SII* Systemic immune-inflammation index, *PLR* Platelet-to-lymphocyte ratio, *NLR* Neutrophil-to-lymphocyte ratio

Bold fonts indicate *P* value < 0.05

^a^Model 1: Unadjusted

^b^Model 2: Adjusted for age, sex, and race

^c^Model 3: Adjusted for age, sex, race, education level, PIR, BMI, smoking status, alcohol consumption status, recreational activities, hypertension, diabetes, CVD and taking immunosuppressants

### Association between sleep-related disorder and SII

The relationships between SII, PLR and NLR and sleep-related disorder were further analyzed by linear regression (Table 3). Overall, sleep-related disorders were found to be more closely associated with SII than the PLR and NLR. Participants had a high prevalence of sleep problems (β: 21.421; 95% CI 1.484, 41.358), OSA symptoms (β: 23.088; 95% CI 0.441, 45.735), and daytime sleepiness (β: 30.320; 95% CI 5.851, 54.789) showed a positive association with higher SII in fully adjusted linear models. In additional analyses of other inflammatory measures, we did not find any association between sleep problems, OSA symptoms, daytime sleepiness, and PLR in this study. However, it is worth mentioning that we did observe higher NLR in participants with daytime sleepiness (β: 0.081; 95% CI 0.002, 0.159).

**Table 3:** Weighted linear regression coefficients (β) and 95% confidence intervals for the association between sleep-related disorder and SII and inflammatory markers: The United States, 2005 to 2008

Exposure	Outcome	Categories	Model 1^a^ β (95% CI), P- value	Model 2^b^ β (95% CI), P- value	Model 3^c^ β (95% CI), P- value
Sleep problems	SII	No	Reference	Reference	Reference
		Yes	**42.557(24.671,60.444)** *P* < 0.0001	**30.607(12.271, 48.942)** *P* = 0.002	**21.421(1.484, 41.358)** *P* = 0.038
	PLR	No	Reference	Reference	Reference
		Yes	**2.967(0.801,5.134)** *P* = 0.009	0.545(-1.620, 2.709) *P* = 0.608	1.776(-0.485, 4.038) *P* = 0.108
	NLR	No	Reference	Reference	Reference
		Yes	**0.083(0.015,0.151)** *P* = 0.019	0.050(-0.018,0.118) *P* = 0.143	0.027(-0.047, 0.102) *P* = 0.421
OSA symptoms	SII	No	Reference	Reference	Reference
		Yes	**36.329(20.009,52.650)** *P* < 0.0001	**38.260(20.259, 56.261)** *P* < 0.001	**23.088(0.441, 45.735)** *P* = 0.046
	PLR	No	Reference	Reference	Reference
		Yes	1.115(-1.208,3.439) *P* = 0.335	0.168(-2.129, 2.464) *P* = 0.881	0.427(-1.894, 2.747) *P* = 0.707
	NLR	No	Reference	Reference	Reference
		Yes	**0.105(0.047,0.163)** *P* < 0.001	0.054(-0.002,0.111) *P* = 0.058	0.033(-0.050, 0.116) *P* = 0.382
Daytime sleepiness	SII	No	Reference	Reference	Reference
		Yes	**45.143(21.344,68.943)** P < 0.001	**39.479(17.142, 61.816)** *P* = 0.001	**30.320(5.851, 54.789)** *P* = 0.021
	PLR	No	Reference	Reference	Reference
		Yes	0.643(-2.772,4.058) *P* = 0.703	0.016(-3.183, 3.216) *P* = 0.992	1.258(-2.300, 4.816) *P* = 0.439
	NLR	No	Reference	Reference	Reference
		Yes	**0.086(0.014,0.157)** *P* = 0.021	**0.107(0.035,0.178)** *P* = 0.005	**0.081(0.002, 0.159)** *P* = 0.046

*Abbreviation: CI*, Confidence interval, *SII* Systemic immune-inflammation index, *PLR* Platelet-to-lymphocyte ratio, NLR Neutrophil-to-lymphocyte ratio, *OSA* Obstructive sleep apnea

Bold fonts indicate *P* value < 0.05

^a^Model 1: Unadjusted

^b^Model 2: Adjusted for age, sex, and race

^c^Model 3: Adjusted for age, sex, race, education level, PIR, BMI, smoking status, alcohol consumption status, recreational activities, hypertension, diabetes, CVD and taking immunosuppressants

### Subgroup and sensitivity analyses

Supplementary Tables 3 and 4 presents the results of our subgroup analyses. The likelihood ratio test for the interactions among the remaining results was not statistically significant, and the main analysis results can be considered stable. Supplementary Tables 5, 6, and 7 display the outcomes of our sensitivity analyses, which consistently yielded similar results when we excluded participants with missing covariates and recent infections. Recognizing that OSA could potentially influence the relationship between inflammation and daytime sleepiness, we conducted additional adjustments, and the findings remained consistent with the primary analysis. In the fully adjusted model, a positive association between daytime sleepiness and higher SII levels in participants was observed (β: 30.320; 95% CI 5.851, 54.789). This association remained significant even after further adjustment for OSA symptoms (β: 26.365; 95% CI 1.801, 50.930), indicating that the results of the model were robust.

## Discussion

To our knowledge, this is the first cross-sectional study to demonstrate that the sleep-related disorders are strongly associated with SII in a nationally representative sample. In this study of 8,505 survey participants, we discovered that sleep problems, a high risk of OSA, and daytime sleepiness were all linked to higher levels of SII, which persisted after adjusting for numerous confounding covariates. Associations were similar across subgroups of subjects defined by age, sex, and race/ethnicity. At the same time, comparable correlations were shown in sensitivity analyses excluded participants with missing covariates and recent infections. Overall, sleep-related disorders were found to have a stronger association with SII compared to PLR and NLR. Additionally, in our analysis of other inflammatory factors, we only observed a positive association between daytime sleepiness and NLR.

To date, the literature on sleep and SII is sparse and confined to special study populations, thus the results of our study complements the literature well. A retrospective cohort study conducted by Topuz's team found a positive association between the SII and OSA prevalence, which increased with disease severity. In addition, that association was stronger than that of the association with NLR and PLR [19]. At the same time, in a prospective study of psychopathology and neurocognitive impairment in COVID-19 survivors, baseline SII levels predicted depressive symptoms such as insomnia and anxiety at the 3-month follow-up [17]. Another study also supports the notion that the systemic inflammatory response, as reflected by the baseline SII, is positively correlated with depression and anxiety scores at follow-up [16]. Recent studies provide additional evidence to support the aforementioned conclusion that inflammatory markers may act as a mediator in the potential influence of sleep disruption on depressive symptoms [30]. A study based on NHANES data examined the relationship between inflammatory markers, sleep disorders, and lifestyle habits. The findings revealed a strong association between leukocyte inflammatory markers, such as SII, and sleep disorders. Additionally, the study found that these inflammatory markers mediated the link between sedentary behavior and sleep disorders [31]. Our findings are consistent with those previously reported in the literature, where subjects with OSA and sleep problems were more likely to have elevated SII levels; however, the results of our study were obtained in nonhospitalized patients and were thus more representative.

The interaction of inflammation and sleep has become an established fact and has been confirmed by increasing research. The inflammatory cytokines interleukin (IL) and tumor necrosis factor (TNF) have been shown to play an important role in mediating inflammation and sleep, have well-established sleep regulatory functions [32, 33] and are dysregulated in sleep-related diseases such as sleep deprivation, circadian misalignment [34] and OSA [35]. Sleep is mediated by the central nervous system to regulate mental psychology and it dynamically regulates the immune system through the production and redistribution of inflammatory cytokines, with the main effectors including the hypothalamic–pituitary–adrenal axis and the sympathetic nervous system [5]. Experimental sleep deprivation alters the circadian rhythm of IL-6 secretion [36], resulting in decreased IL-6 secretion at night and, conversely, increased daytime and correspondingly dysregulated TNF secretion [37]. In a meta-analysis of 72 studies, long sleep duration and sleep disturbance, but not extremely short sleep, were associated with increased C-reactive protein and IL-6 levels [9]. In addition, treatment of sleep disorders such as insomnia can reverse the levels of inflammatory markers and reduce IL-6 and TNF levels to alleviate systemic inflammatory responses [38]. Taken together, the results of these studies show that disturbed sleep may cause damage to host health by overactivating the inflammatory response [39]. At the same time, current perspectives suggest that the relationship between sleep and inflammation works both ways, and disruptions in the body's immune balance may also affect the quality of sleep. An animal study revealed that mice infected with the influenza virus exhibited an increase in non-rapid eye movement sleep, while experiencing a decrease in rapid eye movement sleep. However, the precise role of inflammation in this process remains uncertain [40]. In a sepsis model characterized by significant systemic inflammation, sleep in rats exhibited a fragmented pattern, which was found to be influenced by elevated mRNA and protein levels of cytokines, including IL-1β, IL-6, and TNF-α, in the nervous system [41]. The SII, as a simple and available indicator of the systemic inflammatory response, could play a crucial role in sleep monitoring.

In this study, we are presenting a novel finding that demonstrates a positive correlation between daytime sleepiness and SII. Since daytime sleepiness is prevalent among individuals with OSA [42], we conducted sensitivity analyses to account for this confounding factor by adjusting for OSA symptoms. Interestingly, the results of these analyses indicate that the positive association between daytime sleepiness and SII remains significant. At the same time, it has also been suggested that daytime sleepiness depends on sleep quality and timing and may also be associated with a variety of neuropsychological and cardiopulmonary diseases [43]. A cross-sectional study noted that patients with OSA and excessive daytime sleepiness exhibited elevated levels of high-sensitivity C-reactive protein in people without metabolic syndrome [44]. Another study also showed that objective daytime sleepiness was associated with increased IL-6 levels and decreased cortisol levels and that the disease phenotype of this inflammatory manifestation was associated with cardiometabolic morbidity and mortality [45]. Previous studies have demonstrated a U-shaped relationship between sleep duration and both inflammatory markers and mortality [46, 47]. Similar patterns were observed in this study, but statistically no significant association was found between sleep duration and inflammatory markers such as SII in this study. This finding therefore needs to be interpreted with caution.

The association of SII with sleep-related disorder outcomes carries important public health implications. In the context of the era of COVID-19, we face long-term coexistence with the novel coronavirus. Evidence thus far suggests that up to 50% to 70% of patients with new coronavirus pneumonia are expected to experience severe sleep disorders [48], possibly due to adverse psychological distress caused by the fear of the disease itself among the public affected by social media reports [49]. A single-center retrospective study of the impact of sleep quality on lymphopenia recovery and clinical outcomes in hospitalized patients with COVID-19 found that decreased absolute lymphocyte counts and increased NLR were more pronounced in patients with poor sleep quality [50]. Although our findings are derived from a nonhospitalized national population, it is undeniable that SII, as an indicator that can effectively reflect systemic immune inflammation, will give some inspiration for inflammation and sleep monitoring to subsequent new coronavirus-infected people.

Our study has some distinct strengths. This study represents a national-level population with a sufficiently large sample size. Then, we included a large number of covariates to control for confounding, and the definition of the outcome of sleep-related disorder was consistent with previous studies. However, there are some limitations that need to be noted. The cross-sectional study design does not provide the ability to make causal inferences, and prospective studies are needed to further explore these results. In addition, our combined data are older, but this is also an unavoidable problem, as complete sleep questionnaire data were only recorded in 2005–2008. Furthermore, the data used in this study were extracted from only one hematology test, and no repeat serial testing was performed. Meanwhile, the sleep-related disorders used in this study were based on self-reported data from participants, rather than objective measures like polysomnography or actigraphy. Further research is needed to determine if objective measures of sleep are also linked to SII. Additionally, future studies could explore sleep health as a multidimensional composite analysis, rather than focusing solely on one aspect of sleep health.

## Conclusion

In this study, we discovered that sleep-related disorders had a stronger correlation with SII compared to PLR and NLR. Specifically, we found that sleep problems, a high risk of OSA, and daytime sleepiness were positively linked to SII. Furthermore, our analysis of other inflammatory markers revealed that daytime sleepiness was only associated with NLR. To validate our results, further comprehensive and detailed prospective studies are required.

### Supplementary Information

**Additional file 1:** **Supplementary Table 1.** Definition and Details of Covariates. **Supplementary Table 2.** The numbers and percentages of missing covariate data. **Supplementary Table 3.** Subgroup analysis for the association of sleep duration with SII. **Supplementary Table 4.** Subgroup analysis for the association of sleep-related disorder with SII. **Supplementary Table 5.** Weighted linear regression coefficients (β) and 95% confidence intervals for the association between sleep-related disorder and SII and inflammatory markers: The United States, 2005 to 2008 (exclude participants with missing covariates). **Supplementary Table 6.** Weighted linear regression coefficients (β) and 95% confidence intervals for the association between sleep-related disorder and SII and inflammatory markers: The United States, 2005 to 2008 (exclude participants with recent infection). **Supplementary Table 7.** Weighted linear regression coefficients (β) and 95% confidence intervals for the association between daytime sleepiness and SII and inflammatory markers: The United States, 2005 to 2008 (additional adjustment for OSA symptoms).

## Data Availability

Publicly available datasets were analyzed in this study. This data can be found here: https://wwwn.cdc.gov/nchs/nhanes/.
