# Supplementary material for: Analysis of the relationship between sleep-related disorder and systemic immune-inflammation index in the US population
Source: BMC Psychiatry. 2023 Oct 24;23:773. doi: 10.1186/s12888-023-05286-7 (PMC10594811; doi:10.1186/s12888-023-05286-7)
Supplement: Supplementary file 1 — Additional file 1: Supplementary Table 1. Definition and Details of Covariates. Supplementary Table 2. The numbers and percentages of missing covariate data. Supplementary Table 3. Subgroup analysis for the association of sleep duration with SII. Supplementary Table 4. Subgroup analysis for the association of sleep-related disorder with SII. Supplementary Table 5. Weighted linear regression coefficients (β) and 95% confidence intervals for the association between sleep-related disorder and SII and inflammatory markers: The United States, 2005 to 2008 (exclude participants with missing covariates). Supplementary Table 6. Weighted linear regression coefficients (β) and 95% confidence intervals for the association between sleep-related disorder and SII and inflammatory markers: The United States, 2005 to 2008 (exclude participants with recent infection). Supplementary Table 7. Weighted linear regression coefficients (β) and 95% confidence intervals for the association between daytime sleepiness and SII and inflammatory markers: The United States, 2005 to 2008 (additional adjustment for OSA symptoms). [file 12888_2023_5286_MOESM1_ESM.docx]

Supplementary Material

Analysis of the relationship between sleep-related disorder and systemic immune-inflammation index in the US population

Kaisaierjiang Kadier^1,2†^, Diliyaer Dilixiati^3†^, Aikeliyaer Ainiwaer^1,2†^, Xiaozhu Liu^4^, Jiande Lu^3^, Pengfei Liu^1,2^, Mierxiati Ainiwan^1,2^, Gulinazi Yesitayi^1,2^, Xiang Ma^1,2^* and Yitong Ma^1,2^*

^1^Department of Cardiology, First Affiliated Hospital of Xinjiang Medical University, Urumqi, China

^2^Key Laboratory of Cardiovascular Disease, Xinjiang Clinical Medical Research Institute, Urumqi, China

^3^Department of Urology, First Affiliated Hospital of Xinjiang Medical University, Urumqi, China

^4^Department of Cardiology, The Second Affiliated Hospital of Chongqing Medical University, Chongqing , China.

*** Correspondence:**

Xiang Ma

[maxiangxj@yeah.net](mailto:maxiangxj@yeah.net)

Yitong Ma

myt_xj@sina.com

**^†^Equal contributions:**

^†^These authors have contributed equally to this work and share first authorship

**Supplementary Table 1** Definition and Details of Covariates

**Supplementary Table 2** The numbers and percentages of missing covariate data

**Supplementary Table 3** Subgroup analysis for the association of sleep duration with SII.

**Supplementary Table 4** Subgroup analysis for the association of sleep-related disorder with SII.

**Supplementary Table 5** Weighted linear regression coefficients (β) and 95% confidence intervals for the association between sleep-related disorder and SII and inflammatory markers: The United States, 2005 to 2008 (exclude participants with missing covariates)

**Supplementary Table 6** Weighted linear regression coefficients (β) and 95% confidence intervals for the association between sleep-related disorder and SII and inflammatory markers: The United States, 2005 to 2008 (exclude participants with recent infection)

**Supplementary Table 7** Weighted linear regression coefficients (β) and 95% confidence intervals for the association between daytime sleepiness and SII and inflammatory markers: The United States, 2005 to 2008 (additional adjustment for OSA symptoms)

**Supplementary Table 1** Definition and Details of Covariates

| **Covariate** | **Definition and NHANES codon** | **Grouping for adjustment** |
| --- | --- | --- |
| **Age** | Age at Screening (RIDAGEYR) | Continuous Variables |
| **Sex** | Gender (RIAGENDR) | Male; Female |
| **Race** | Race/Ethnicity (RIDRETH1) | Non-Hispanic White; Non-Hispanic Black; Mexican American; Other Hispanic; Other race/ethnicity |
| **Education level** | Education Level - Adults 20+ (DMDEDUC2) | Less than high school; High school grad; Above high school |
| **Poverty-income ratio** | A ratio of family income to poverty threshold (INDFMPIR) | <1.3; 1.3-3.5; >3.5 |
| **Body mass index** | Body Mass Index (kg/m^2^) (BMXBMI) | <30; ≥30 |
| **Smoking status** | Never smokers: smoking less than 100 cigarettes in their life. Former smokers: smoked more than 100 cigarettes in their life and had quit smoking. Current smokers smoked more than 100 cigarettes in their life and smoked some days or every day. (SMQ020; SMQ040) | Current smokers; Former smokers; Never smokers |
| **Alcohol consumption status** | Never drinkers: had <12 drinks in lifetime. Former drinkers: had ≥12 drinks in 1 year and did not drink last year, or did not drink last year but drank ≥12 drinks in lifetime. Current mild-moderate drinkers: ≤1 drink per day for women or ≤2 drinks per day for men on average over the past year. Current heavier drinkers: >1 drink per day for women or >2 drinks per day for men on average over the past year. (ALQ101; ALQ110; ALQ120Q) | Never drinkers; Former drinkers; Current mild-moderate drinkers; Current heavier drinkers |
| **Recreational activitie** | Vigorous: over the past 30 days, do any vigorous activities for at least 10 minutes. Moderate: over the past 30 days, do any Moderate activities for at least 10 minutes. Inactive: the above questions were negative responses. (PAQ650; PAQ665; PAD200; PAD320) | Vigorous; Moderate; Inactive |
| **Hypertension** | Diagnosis by a doctor or other health professional, average blood pressure ≥130/80 mmHg or use of hypertension medication. (BPQ020; BPXSY1; BPXDI1; BPXSY2; BPXDI2; BPXSY3; BPXDI3; BPQ040A) | Yes; No |
| **Diabetes** | Diagnosis by a doctor or other health professional, glycohemoglobin (%) >6.5, random blood glucose (mmol/l) ≥11.1, or use of diabetes medication or insulin. (DIQ010; DIQ050; DIQ070; LBXGH; LBDSGLSI) | Yes; No |
| **Cardiovascular disease** | Diagnosis by a doctor or other health professional, including congestive heart failure, angina pectoris, coronary heart disease, heart attack, or stroke. (MCQ160B; MCQ160C; MCQ160D; MCQ160E; MCQ160F) | Yes; No |

NHANES, National Health and Nutrition Examination Survey.

**Supplementary Table 2** The numbers and percentages of missing covariate data

| **Covariate** | **Numbers** | **Percentages (%)** |
| --- | --- | --- |
| Education | 6 | 0.07 |
| Poverty-income ratio | 568 | 6.68 |
| Body mass index | 129 | 1.52 |
| Smoking status | 5 | 0.06 |
| Alcohol consumption status | 610 | 7.17 |
| Recreational activities | 1 | 0.01 |
| Cardiovascular diseases | 1 | 0.01 |
| Hypertension | 2 | 0.02 |
| Taking immunosuppressants | 7 | 0.08 |

**Supplementary Table 3** Subgroup analysis for the association of sleep duration with SII.

| Subgroup | Categories | Fully adjusted model^a^  β (95% CI), P- value | P for interaction^b^ |
| --- | --- | --- | --- |
| **Age** |  |  | 0.345 |
| <60 year | 7–9 h/night | Reference |  |
|  | < 7 h/night | -6.345( -26.597, 13.907)  P=0.491 |  |
|  | > 9 h/night | 37.667( -44.356,119.691)  P=0.321 |  |
| ≥60 year | 7–9 h/night | Reference |  |
|  | < 7 h/night | 32.135( -9.272, 73.542)  P=0.111 |  |
|  | > 9 h/night | 21.587( -84.461,127.635)  P=0.651 |  |
| **Sex** |  |  | 0.794 |
| Male | 7–9 h/night | Reference |  |
|  | < 7 h/night | 0.515( -27.698, 28.728)  P=0.967 |  |
|  | > 9 h/night | 42.783( -59.744,145.310)  P=0.364 |  |
| Female | 7–9 h/night | Reference |  |
|  | < 7 h/night | 3.171( -26.119, 32.460)  P=0.809 |  |
|  | > 9 h/night | 31.222( -65.578,128.022)  P=0.478 |  |
| **Race** |  |  | 0.556 |
| Non-Hispanic white | 7–9 h/night | Reference |  |
|  | < 7 h/night | 1.387( -24.040, 26.814)  P=0.906 |  |
|  | > 9 h/night | 55.719( -21.840,133.277)  P=0.141 |  |
| Non-Hispanic black | 7–9 h/night | Reference |  |
|  | < 7 h/night | 21.224( -14.311, 56.759)  P=0.206 |  |
|  | > 9 h/night | 41.967( -59.788, 143.723)  P=0.369 |  |
| Other races^c^ | 7–9 h/night | Reference |  |
|  | < 7 h/night | -7.145( -41.571, 27.280)  P=0.657 |  |
|  | > 9 h/night | -26.058(-138.222, 86.107)  P=0.619 |  |

Abbreviation: SII, systemic immune-inflammation index; CI, confidence interval.

^a^ Adjusted for age, sex, race, education level, PIR, BMI, smoking status, alcohol consumption status, recreational activities, hypertension, diabetes, CVD and taking immunosuppressants.

^b^ P for interaction were assessed using likelihood ratio tests.

^c^ Other races included Mexican American, other Hispanic and other multi-racial.

**Supplementary Table 4** Subgroup analysis for the association of sleep-related disorder with SII.

| Exposure | Subgroup | Fully adjusted model^a^  OR (95% CI), P- value | P for interaction^b^ |
| --- | --- | --- | --- |
| Sleep problems | **Age** |  | 0.976 |
|  | <60 year | **22.342( 2.313, 42.370)**  **P=0.033** |  |
|  | ≥60 year | 21.409( -20.967, 63.784)  P=0.283 |  |
|  | **Sex** |  | 0.457 |
|  | Male | 13.495( -15.734, 42.723)  P=0.324 |  |
|  | Female | 28.865( -0.864, 58.594)  P=0.056 |  |
|  | **Race** |  | 0.416 |
|  | Non-Hispanic white | **25.621( 1.016, 50.226)**  **P=0.043** |  |
|  | Non-Hispanic black | 18.679( -15.954, 53.312)  P=0.253 |  |
|  | Other races^c^ | 6.893( -26.139, 39.926)  P=0.657 |  |
| OSA symptoms | **Age** |  | 0.544 |
|  | <60 year | 19.527( -4.145, 43.199)  P=0.097 |  |
|  | ≥60 year | 43.68( -9.276, 96.636)  P=0.098 |  |
|  | **Sex** |  | 0.096 |
|  | Male | 3.328(-23.370, 30.027)  P=0.791 |  |
|  | Female | **45.665( 11.063, 80.267)**  **P=0.014** |  |
|  | **Race** |  | 0.463 |
|  | Non-Hispanic white | 23.802( -3.521, 51.126)  P=0.083 |  |
|  | Non-Hispanic black | 29.621( -8.798, 68.041)  P=0.119 |  |
|  | Other races^c^ | 18.665( -28.440, 65.771)  P=0.412 |  |
| Daytime sleepiness | **Age** |  | 0.144 |
|  | <60 year | 19.704( -5.368, 44.776)  P=0.109 |  |
|  | ≥60 year | 60.665( -0.697,122.028)  P=0.052 |  |
|  | **Sex** |  | 0.812 |
|  | Male | **33.17( 2.791, 63.549)**  **P=0.036** |  |
|  | Female | 27.37( -3.208, 57.947)  P=0.074 |  |
|  | **Race** |  | 0.199 |
|  | Non-Hispanic white | **41.454( 13.996, 68.912)**  **P=0.007** |  |
|  | Non-Hispanic black | 13.446( -19.622, 46.514)  P=0.382 |  |
|  | Other races^c^ | -6.99( -54.214, 40.233)  P=0.753 |  |

Abbreviation: SII, systemic immune-inflammation index; CI, confidence interval; OSA: obstructive sleep apnea.

Bold fonts indicate P value < 0.05.

^a^ Adjusted for age, sex, race, education level, PIR, BMI, smoking status, alcohol consumption status, recreational activities, hypertension, diabetes, CVD and taking immunosuppressants.

^b^ P for interaction were assessed using likelihood ratio tests.

^c^ Other races included Mexican American, other Hispanic and other multi-racial.

**Supplementary Table 5** Weighted linear regression coefficients (β) and 95% confidence intervals for the association between sleep-related disorder and SII and inflammatory markers: The United States, 2005 to 2008 (exclude participants with missing covariates)

| Exposure | Outcome | Categories | Fully adjusted model^a^  β (95% CI), P- value |
| --- | --- | --- | --- |
| Sleep problems | SII | No | Reference |
|  |  | Yes | **24.304( 1.252, 47.356)**  **P=0.041** |
|  | PLR | No | Reference |
|  |  | Yes | 2.187( -0.483, 4.857)  P=0.096 |
|  | NLR | No | Reference |
|  |  | Yes | 0.023(-0.059, 0.105)  P=0.534 |
| OSA symptoms | SII | No | Reference |
|  |  | Yes | **27.185( 3.906, 50.464)**  **P=0.026** |
|  | PLR | No | Reference |
|  |  | Yes | 0.426( -1.896, 2.749)  P=0.707 |
|  | NLR | No | Reference |
|  |  | Yes | 0.052(-0.034, 0.139)  P=0.199 |
| Daytime sleepiness | SII | No | Reference |
|  |  | Yes | **33.442( 6.110, 60.774)**  **P=0.022** |
|  | PLR | No | Reference |
|  |  | Yes | 1.397( -2.482, 5.276)  P=0.430 |
|  | NLR | No | Reference |
|  |  | Yes | 0.085(-0.008, 0.178)  P=0.068 |

Abbreviation: CI, confidence interval; SII, systemic immune-inflammation index; PLR, platelet-to-lymphocyte ratio; NLR, neutrophil-to-lymphocyte ratio; OSA: obstructive sleep apnea.

Bold fonts indicate P value < 0.05.

^a^ Adjusted for age, sex, race, education level, PIR, BMI, smoking status, alcohol consumption status, recreational activities, hypertension, diabetes, CVD and taking immunosuppressants.

**Supplementary Table 6** Weighted linear regression coefficients (β) and 95% confidence intervals for the association between sleep-related disorder and SII and inflammatory markers: The United States, 2005 to 2008 (exclude participants with recent infection)

| Exposure | Outcome | Categories | Fully adjusted model^a^  β (95% CI), P- value |
| --- | --- | --- | --- |
| Sleep problems | SII | No | Reference |
|  |  | Yes | **25.494( 1.405, 49.583)**  **P=0.041** |
|  | PLR | No | Reference |
|  |  | Yes | 1.958( -0.539, 4.454)  P=0.108 |
|  | NLR | No | Reference |
|  |  | Yes | 0.056(-0.027, 0.139)  P=0.161 |
| OSA symptoms | SII | No | Reference |
|  |  | Yes | 24.538( -0.610, 49.687)  P=0.055 |
|  | PLR | No | Reference |
|  |  | Yes | 1.016( -1.787, 3.819)  P=0.460 |
|  | NLR | No | Reference |
|  |  | Yes | 0.037(-0.053, 0.128)  P=0.368 |
| Daytime sleepiness | SII | No | Reference |
|  |  | Yes | **28.547( 4.216, 52.878)**  **P=0.027** |
|  | PLR | No | Reference |
|  |  | Yes | 1.322( -2.688, 5.331)  P=0.469 |
|  | NLR | No | Reference |
|  |  | Yes | **0.09( 0.005, 0.175)**  **P=0.040** |

Abbreviation: Recent infection is defined as any illness within the last 30 days that includes symptoms such as a head or chest cold, stomach or intestinal disease with vomiting or diarrhea, influenza, pneumonia, or an ear infection.

CI, confidence interval; SII, systemic immune-inflammation index; PLR, platelet-to-lymphocyte ratio; NLR, neutrophil-to-lymphocyte ratio; OSA: obstructive sleep apnea.

Bold fonts indicate P value < 0.05.

^a^ Adjusted for age, sex, race, education level, PIR, BMI, smoking status, alcohol consumption status, recreational activities, hypertension, diabetes, CVD and taking immunosuppressants.

**Supplementary Table 7** Weighted linear regression coefficients (β) and 95% confidence intervals for the association between daytime sleepiness and SII and inflammatory markers: The United States, 2005 to 2008 (additional adjustment for OSA symptoms)

| Exposure | Outcome | Categories | Fully adjusted model^a^  β (95% CI), P- value |
| --- | --- | --- | --- |
| Daytime sleepiness | SII | No | Reference |
|  |  | Yes | **26.365( 1.801, 50.930)**  **P=0.039** |
|  | PLR | No | Reference |
|  |  | Yes | 0.358( -3.422, 4.137)  P=0.829 |
|  | NLR | No | Reference |
|  |  | Yes | **0.077( 0.001, 0.153)**  **P=0.048** |

Abbreviation: CI, confidence interval; SII, systemic immune-inflammation index; PLR, platelet-to-lymphocyte ratio; NLR, neutrophil-to-lymphocyte ratio; OSA: obstructive sleep apnea.

Bold fonts indicate P value < 0.05.

^a^ Adjusted for age, sex, race, education level, PIR, BMI, smoking status, alcohol consumption status, recreational activities, hypertension, diabetes, CVD, taking immunosuppressants and OSA symptoms.
